# *Terfezia boudieri*: A Desert Truffle With Anticancer and Immunomodulatory Activities

**DOI:** 10.3389/fnut.2020.00038

**Published:** 2020-04-08

**Authors:** Maha Farid Al Obaydi, Wafaa M. Hamed, Lina T. Al Kury, Wamidh H. Talib

**Affiliations:** ^1^Department of Clinical Pharmacy and Therapeutic, Applied Science Private University, Amman, Jordan; ^2^Pharmacy Department, AlNoor University College, Mosul, Iraq; ^3^Department of Health Sciences, College of Natural and Health Sciences, Zayed University, Abu Dhabi, United Arab Emirates

**Keywords:** truffles, fungi, anticancer, functional food, immunomodulatory

## Abstract

Desert truffles have high nutritional value and grow wild in the Mediterranean basin and Western Asia. Although, many studies were performed to evaluate truffles nutritious values and phytochemical composition, studies are limited to evaluate their anticancer and/ or immunomodulatory effects. Our study was conducted to evaluate the anticancer and immunomodulatory effects of *Terfezia boudieri* (desert truffle). Different solvent extracts were prepared from the truffle and MTT assay was used to measure their anticancer activity against cancer cell lines (T47D, MCF-7, MDA-MB231, HCT-116, and Hela). Total phenolic content in each extract was determined by using Folin-Ciocalteu reagent and qualitative phytochemical screening was performed using standard methods. The degree of apoptosis induction (using caspase 3 assay) and vascular endothelial growth factor expression were detected using standard kits. Also, ELISA was used to measure levels of IFN-γ, IL-2, IL-4, and IL-10 secreted by splenocytes after treatment with the extracts. The effect of the extracts on splenocytes proliferation was measured using MTT assay. Macrophage function was evaluated using nitro blue tetrazolium assay and pinocytosis function was evaluated using neutral red method. Terpenoids, phytosterols, and carbohydrates were present in all the solvent extracts, while tannins, alkaloids and flavonoids were detected only in aqueous/methanol and aqueous extracts. The highest total phenolic content was observed in aqueous and aqueous methanol extracts. The growth of cancer cell lines was inhibited by *T. boudieri* extracts in a dose dependent manner. N-hexane extract was the most potent against most cell lines. Aqueous/methanol extract showed high apoptosis induction and angiogenesis suppression effects. An increase in TH1 cytokines (IFN-γ, IL-2) level and a decrease in TH2 cytokine (IL-4) level were evident after lymphocytes stimulation by aqueous/methanol, n-hexane and ethyl acetate extracts of *T. boudieri*. Ethyl acetate extract of *T. boudieri* were the most potent extracts to stimulate lymphocytes proliferation while all other extracts showed moderate stimulation. Aqueous/methanol extract was the most active extract to stimulate phagocytosis. Ethyl acetate extract was the most active extract to stimulate pinocytosis. The use of *T. boudieri* provides variable health benefits. N-hexane, ethyl acetate, and aqueous/methanol extracts exhibited anticancer activities and are potent stimulators of innate and acquired immunity. Further testing is needed to identify the biologically active compounds and detect them quantitatively using GC-MS analysis.

## Introduction

Cancer is a global health problem and number of cancer patients is in continuous rise (1). Approximately, and according to World Health Organization (WHO), there were 14.1 million new cancer cases and expected to rise to 22 million within the next two decades. Additional statistics showed that 70% of deaths from cancer occur in low and middle income countries (2). Therefore, cancer became a heavy problem worldwide (3). The economic impact of cancer is significant and is increasing. The total annual economic cost of cancer treatment in 2010 was estimated at approximately US$ 1.16 trillion (4).

Diet is one of the most important factors for the formation and prevention of cancer, the link between them is just as mysterious as the disease itself (5). Researches have pointed to certain nutrients and foods that may help contribute or, conversely, prevent certain types of cancer (6). High intakes of fruit and vegetables with high antioxidants and fibers tend to reduce the risk of cancer at several sites. Evidences have been derived the relationship between consumption of vegetables and fruits and the risk of several common cancers (7). Total intake of calories seems to have a strong positive influence on causing cancer, and the increasing in breast, colon, and prostate cancer incidence is associated with consumption of fat-rich food (8).

The use of conventional anticancer therapies (chemotherapy and radiation) is associated with serious side effects (9). These side effects encourage scientists exploring alternative cancer therapies to enhance the efficiency of current therapies and reduce toxicity. An attractive source for these therapies was the natural products (10). Among them were the edible medicinal fungi which produce medically significant metabolites or can be induced to produce such metabolites using biotechnology (11). These fungi may inhibit cancer cell by augmenting the function of the immune system (12).

About 82,000 fungal species were discovered and yet to be (13). Macrofungi can be classified into two types: epigeous (mushrooms) and hypogeous (truffles) (14). Modern medicine rediscovered ancient super foods, as a growing evidence that mushrooms (such as White button, Shiitake, Maitake, portabella, Reishi, Turkey Tail) are among the potentially positive foods for cancer fighting and prevention (15). Previous research on *Terfezia boudieri* reported a remarkable antibacterial, antioxidant, and radical scavenging activities of flavonoids rich extract of this truffle (16, 17).

In spite of the fact that recent truffles research emphasis on the chemical properties (nutritional and aromatic profile) and their potential biological activities, further scientific studies need to pay greater attention to the value added to truffles.

This study was executed to investigate the benefits of *Terfezia boudieri* extracts as a source of immunomodulatory and anticancer agents.

## Materials and Methods

### Cell Lines and Cell Culturing Condition

Six cell lines were used to investigate the anticancer effect of *T. boudieri* extracts. The cells were cultured in complete medium and incubated at 37°C in 5% CO_2_, 95% humidity incubator. Two human epithelial breast cancer cell lines (T47D and MCF-7), human breast adenocarcinoma cell line (MDA-MB-231), human colon carcinoma cell line (HCT-116), and human epitheloid cervix carcinoma cell line (Hela) were used in this study. T47D and MCF-7 cell lines were cultured in complete RPMI 1640 medium. MDA-MB231, HCT-116, and Hela cell lines were cultured in complete DMEM medium with high glucose. Kidney epithelial cells from African green monkey (Vero) were used as normal control and cultured in complete DMEM medium. All culture media were supplemented with 1% L-glutamine, 10% fetal bovine serum, 1% penicillin-streptomycin, and 0.1% gentamycin solution.

### Truffles Collection and Extracts Preparation

Truffles (*T. boudieri*) were purchased from Jordanian market, and identified by Mr. Anas AbuYahya (monitoring and evaluation specialist/ flora researcher, in the Royal Society for the Conservation of Nature, Amman- Jordan). Fresh truffles were cleaned well (by brush followed by wet towel), sliced (to the thickness of about 1–3 mm), dried, powdered and kept in dry and dark place. Different extracts using solvents of different polarities were prepared from 250 g of the powdered truffle material. Aqueous/methanol (80:20%) extract was prepared by macerating the dried powder (250 g) at room temperature for 3 days. Then the extract was filtered and the supernatant was concentrated using rotary evaporator. Complete drying of the extract was achieved using lyophilizer, and then the extract was kept at −20°C until used. Water, n-hexane and ethyl acetate extracts were prepared by dissolving of aqueous/methanol extract (1.5 g) in water, then fractionating it using n-hexane, followed by ethyl acetate. Both fractions were concentrated using rotary evaporator. The remaining water fraction was dried completely and all extracts was stored at −20°C until used.

### Phytochemical Screening

Phytochemical examinations were carried out for all *T. boudieri* extracts. A qualitative chemical screening for identification of various classes of active chemical constituents such as saponins, tannins, phytosterols, terpenoids, alkaloids, flavonoids, anthraquinones, and carbohydrates were performed. Detection of the extracted compounds was done according to the standard methods described by Trease and Evans (18).

### Total Phenolic Content (TPC) by Folin-Ciocalteu Method

The amount of TPC in *T. boudieri* extracts was determined according to the F-C procedure described by Akyüz (17). A stock solution of 25 mg/ml of each extract was prepared. Five different dilutions were further made from each stock as 20, 15, 10, 5, 2.5 mg/ml. Briefly, 12.5 μl of each dilution was mixed with 250 μl of 2% sodium carbonate solution in 96-well microplate in duplicate. They were allowed to react for 5 min at room temperature (RT). Then, 12.5 μl of 50% F-C reagent was added and allowed to stand again for 30 min at RT. The absorbance of reaction mixture was read using a plate reader at 630 nm. A standard curve was obtained using gallic acid standard solution at various concentrations ranging from 0.1 to 1.0 mg/ml in distilled water. Total phenolic content (mg/ml) of each extract was obtained the standard curve of gallic acid. Data are expressed as equivalent of gallic acid (mg) for each milliliter of each extract (19).

## Antiproliferative Assay

Cells (from different cell lines) were dispensed (100 μl/well) into 96-well tissue culture plates (flat bottom) at an optimized concentration of 15,000 cells/well in complete tissue culture medium. After 24 h, the media in each well were completely removed and the attached cells were treated in triplicates with decreasing concentrations of different extracts (initially dissolved in DMSO) of *T. boudieri* (25–0.78 mg/ml. Plates were incubated for 48 h, and then cell viability was measured by using MTT [3-(4,5-Dimethylthiazol-2-yl)-2,5-diphenyltetrazolium bromide] assay kit (Sigma, USA) assay as previously described (20). Percentage survival was calculated by comparing the absorbance of treated cells to that of untreated cells (tissue culture media + 0.1% DMSO). The concentration at which there was 50% of cell death (IC_50_) was calculated using non-linear regression in SPSS.

### Determination of VEGF Expression in T47D Cells

T47D cells were dispensed into four separated tissue culture flasks at a concentration of 150,000 cells/ml of complete tissue culture medium. After 24 h, the media in each flask was completely removed and the attached cells were subjected to one of the following treatments: aqueous/methanol extract at a concentration of 12.5 mg/ml, 250 nM of doxorubicin, and a negative control (tissue culture media + 0.1% DMSO). Cells were incubated for 48 h, after that the media of each flask were transferred into sterile tubes and VEGF levels were measured using VEGF ELISA kit (Sigma-Aldrich, St. Louis, MO, USA) as previously described (21). A standard curve was obtained using mouse VEGF at various concentrations. The concentration of VEGF for each treatment was obtained using VEGF standard curve. Data are expressed as equivalent of VEGF (pg/ml) for each milliliter of the extract.

### Apoptosis Detection in T47D Cells

T47D cells were dispensed into four separated tissue culture flasks at a concentration of 150,000 cells/ml of complete tissue culture medium. After 24 h, the media in each flask was removed and the attached cells were treated with one of the following treatments: aqueous/methanol extract at a concentration of 25 mg/ml, 250 nM of doxorubicin hydrochloride, and a negative control. Cells were incubated for 48 h with different treatments, after that the media of each flask were removed and the attached cells were harvested. Caspase-3 activity was measured using kit instructions (Sigma-Aldrich, St. Louis, MO, USA). Fold-increase in Caspase 3 activity was determined by comparing extracts results with the level of the negative control. Detailed and step by step procedure was conducted as described previously (22).

### Mice

Balb/C female mice were used in this study. Mice were 4–6 weeks old with average body weight of 21–25 g/mouse. All animal procedures were studied and approved by the Research and Ethical Committee of Applied Science University (Approval Number: 2017-PHA-9). Separate cages with bedding of wood shaving were used to house mice. The environmental conditions of the animal house were stable temperature at 25°C, 50–60% humidity, continuous air ventilation, and alternating light/dark cycles of 12 h.

### Preparation of Murine Splenocytes

Balb/C mouse was sacrificed and the spleen was removed aseptically. The cells of the spleen (splenocytes) were freed by passing spleen tissue through a mesh of a tissue grinder. A suspension of splenocytes was prepared in RPMI-1640. The cell suspension was washed three times using RPMI-1640 and then re-suspended in 5 ml red blood cells lysis buffer (1 mol/L NH4Cl). After 10 min the cells were again centrifuged and re-suspended in RPMI-1640 media. Splenocytes were washed and cell viability was detected using trypane blue assay. Different densities of cell suspension were counted and used in other assays.

### Determination of Cytokines Levels in Activated Lymphocytes

Levels of IFN-γ, IL-2, IL-4, and IL-10 were measured for splenocytes cultured for 48 h with different extracts of *T. boudieri* using mouse TH1/TH2 ELISA kit (affymetrix ebioscience, Canada). Briefly, splenocytes suspension were made (2 × 10^6^ cells/ml) in complete RPMI-1640 and were seeded into 96-well culture plate, 100 μl of different truffle extracts (25 mg/ml in RPMI-1640) were added (triplicate), and the plate was incubated for 48 h CO_2_ incubator. After incubation, culture supernatants were collected to estimate the concentrations of IFN-γ, IL-2, IL-4, and IL-10 using standard kit as previously described (23). A standard curve was prepared using known concentrations of each cytokine. The absorbance values obtained for each treatment were converted into concentration (pg/ml) using standard curve.

### Lymphocytes Proliferation Assay

This assay was performed using MTT [3-(4, 5-Dimethylthiazol-2-yl)-2, 5-diphenyltetrazolium bromide] assay kit (Sigma, USA) according to the manufacturer's instructions. Briefly, splenocytes suspension were made (2 × 10^6^ cell/ml) in complete RPMI-1640 and were seeded into 96-well culture plate in the presence of 5 μg/ml Con A or 4μg/ml LPS. To this, 100 μl of increasing concentrations (5–25 mg/mL in RPMI-1640) of *T. boudieri* extracts were added (triplicate). The plate was incubated for 48 h under 5% CO_2_ and humidified atmosphere of 95% air at 37°C temperature. After the incubation, 10 μl MTT (5 mg/ml) solution was added to each well. The plate was wrapped with aluminum foil to avoid exposure to light and incubated for 4 h. Then 100 μl DMSO was added to each well to dissolve the formazan particles and the absorbance was measured at 550 nm using ELISA microplate reader. Results were expressed as a percentage of proliferation (%) compared to the negative control cells (24). Exactly the same procedure was repeated with the exclusion of the addition of Con A and LPS.

### Macrophage Isolation From Peritoneal Fluid

Forty-eight hours before collection of peritoneal macrophages (PEM), mice were injected intra-peritoneally injected with 1 mL of 6% starch broth medium. Mice were euthanized by cervical dislocation and their abdominal cavities were visualized then 5 ml ice-cold PBS was introduced into the cavity. After gentle massaging the fluid was withdrawn and placed in a centrifuge. After centrifugation of the pooled fluid, cell pellet was suspended in complete RPMI 1640 medium and allowed to adhere for 3 h at 37°C in 5% CO_2_ humidified incubator. Thereafter, non-adherent cells were washed away with medium and the adherent cells then collected and counted to be used in the various assays outlined below (25).

## *In vitro* Phagocytic Assay [Nitro Blue Tetrazolium (NBT) Reduction Test]

The phagocytic activity of macrophage was evaluated using NBT reduction assay. This assay was carried out according to the method previously described by Rainard (26). In brief, PEM (5 × 10^6^ cells/well of a 96-well plate) were cultured with different concentrations of *T. boudieri* extracts (25–3.125 mg/ml) for 48 h. Thereafter, 20 μl yeast suspension (5 × 10^7^ cells/ml in PBS) and 20 μl nitro blue tetrazolium (NBT) (1.5 mg/ml in PBS) were added to each well. Wells that received 20 μl PBS and 20 μl DMSO were used as negative controls. Cells were then incubated for 60 min at 37°C, and the supernatant was then removed and the adherent macrophages were rinsed with RPMI 1640. The cells were air-dried before 120 μl of 2M KOH and 140 μl DMSO were added to each well. The absorbance of the turquoise blue solution was measured at 570 nm (OD570) in the plate reader. The percentage of NBT reduction (reflects phagocytic activity) was calculated as following equation (24):

$$\begin{array}{l} \text{Phagocytic index}=\left(\text{OD sample}-\text{OD control}\right)/\\ \text{ OD contro}{\text{l}}^{*}\;100 \end{array}$$

## Pinocytic Activity Assay by Neutral Red Method

Peritoneal mice macrophages were collected and cultured for 48 h with variant concentrations of *T. boudieri* extracts (25–3.125 mg/ml) using 96-well plate. One hundred microliter of neutral red solution (7.5 mg/ml in PBS) were added to each well and incubated for 2 h. The supernatant was discarded and cells in 96-well plate were washed with PBS twice to remove the neutral red that was not pinocytized by macrophage. Then, 100 μl of cell lysis solution (ethanol and 0.01% acetic acid at the ratio of 1:1) were added to each well to lyse cells. After the incubation of cells at room temperature overnight, the optical density was measured at 540 nm. Pinocytic activity was expressed in terms of absolute OD values (reflecting dye uptake) (24).

## Statistical Analysis

Data are presented using mean ± SEM (Standard Error of Mean). The statistical significance among the groups was determined by one-way analysis of variance (ANOVA) using SPSS (Statistical Package for the Social Science, Chicago, Illinois). A *p* < 0.05 was considered significant. The IC_50_ was obtained for the different extracts of *T. boudieri* using non-linear regression in SPSS (version 21).

## Results

### Aqueous and Aqueous/Methanol Extracts Produced the Highest Yield and Contain Alkaloids and Flavonoids

High variations in percentage yield were observed upon the extraction of 250 gm of *T. boudieri*, using different extraction solvents (Table 1). By using maceration method, the percentage yield reported for *T. boudieri* aqueous/methanol extract was (8.16%). Then by fractionation method for *T. boudieri* aqueous/methanol extract using water, n-hexane, and ethyl acetate solvents, the highest yield was reported for aqueous extract (64.7%), while the lowest yield was reported for ethyl acetate extract (0.4%).

**Table 1:** The percentage yield obtained from the extraction of 250 g of *T. boudieri* using different extraction solvents.

	**Extraction method**	**Extraction solvent**	**% of dried extracts yields**
	Maceration	Aqueous/methanol	8.16
	Fractionation	Water	64.7
*T. boudieri*		n-hexane	1.2
		Ethyl acetate	0.4

The qualitative phytochemical screening results revealed that terpenoids, phytosterols, and carbohydrates were present in all the solvent extracts, while saponins and anthraquinones were absent in all extracts. Tannins, alkaloids, and flavonoids were detected only in aqueous/methanol and aqueous extracts (Table 2).

**Table 2:** Phytochemical screening results obtained from the extraction of *T. boudieri* using different extraction solvents (a concentration of 50 mg/ml was used for each extract).

Phytochemical screening tests^*^	***T. boudieri***			
	**Aqueous/methanol**	**Aqueous**	**n-hexane**	**Ethyl acetate**
Saponins	**–**	**–**	**–**	**–**
Tannins	**+++**	**++**	**–**	**–**
Terpenoids	**+**	**++**	**++**	**+++**
Phytosterols	**+**	**++**	**++**	**+++**
Alkaloids	**++**	**+++**	**–**	**–**
Flavonoids	**+**	**++**	**–**	**–**
Anthraquinones	**–**	**–**	**–**	**–**
Carbohydrates	**++**	**+++**	**++**	**+**

* *Results were rated as: ^+^weak positive; ^++^moderate positive; ^+++^strong positive; –, negative*.

### N-Hexane and Ethyl Acetate Extracts Showed the Highest Antiproliferative Activity Against All Cell Lines

**Figure 1:**
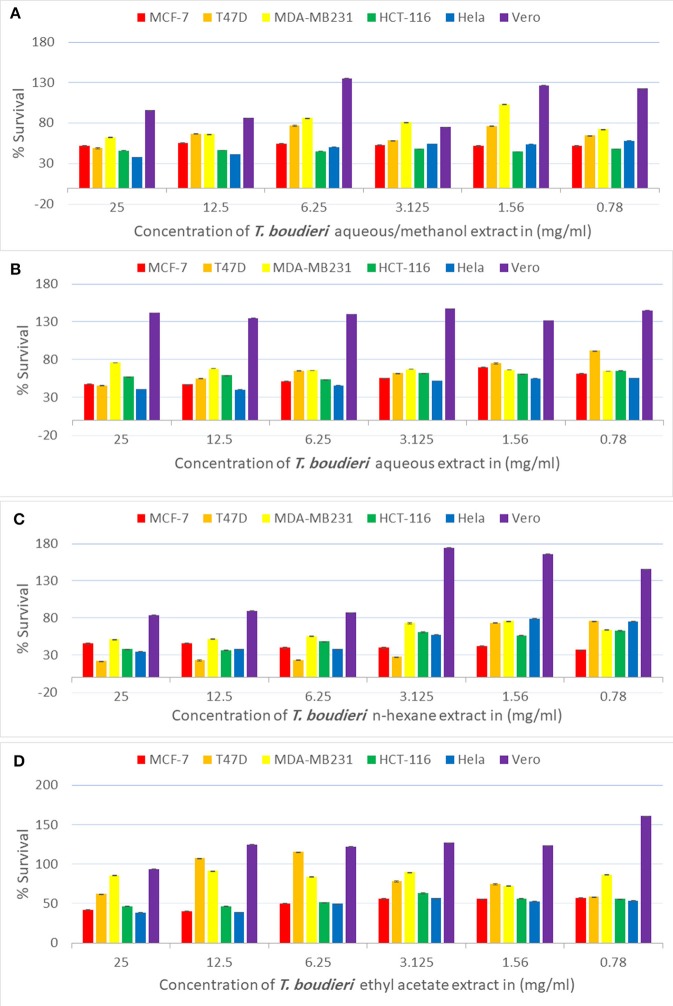
**(A)** Antiproliferative activity of aqueous/methanol extract of *T. boudieri* on MCF-7, T47D, MDA-MB231, HCT-116, Hela and Vero cell lines. **(B)** Antiproliferative activity of aqueous extract of *T. boudieri* on MCF-7, T47D, MDA-MB231, HCT-116, Hela and Vero cell lines. **(C)** Antiproliferative activity of n-hexane extract of *T. boudieri* on MCF-7, T47D, MDA-MB231, HCT-116, Hela and Vero cell lines. **(D)** Antiproliferative activity of ethyl acetate extract of *T. boudieri* on MCF-7, T47D, MDA-MB231, HCT-116, Hela and Vero cell lines. Results are expressed as means of three independent experiments (bars) ± SEM (lines).

Testing decreasing concentrations of *T. boudieri* extracts (25–0.78 mg/ml) on MCF-7 cell line resulted in a dose-dependent manner. The inhibition percentages of queous/methanol, aqueous, n-hexane and ethyl acetate extracts were 49, 43, 54, and 52%, respectively (Figures 1A–D) at concentration of 25 mg/ml for each extract. N-hexane and ethyl acetate extracts showed the highest activity with IC_50_ values of <0.78 mg/ml, while aqueous/methanol and aqueous extracts were the least effective against MCF-7 cell line with IC_50_ values more than 25 mg/ml (Table 3).

**Table 3:** The IC_50_ (mg/ml) for different extracts of *T. boudieri* tested on six different cell lines (MCF-7, T47D, MDA-MB231, HCT-116, Hela, and Vero).

**Cell line**	***T. boudieri***			
	**Aqueous/methanol (mg/ml)**	**Aqueous (mg/ml)**	**n-hexane (mg/ml)**	**Ethyl acetate (mg/ml)**
MCF-7	>25	>25	<0.78	<0.78
T47D	8.5	13	<0.78	8
MDA-MB231	>25	>25	3.75	>25
HCT-116	<0.78	>25	2	1.5
Hela	11.5	2	2	1
Vero	>25			

For T47D cells, the inhibition percentages of aqueous/methanol, aqueous, n-hexane and ethyl acetate extracts were 69, 61, 61, and 71%, respectively (Figures 1A–D). at concentration of 25 mg/ml. Aqueous/methanol, aqueous, n-hexane and ethyl acetate extracts showed high activity against T47D cell line with IC_50_ values of 8.5, 13, <0.78, and 8 mg/ml, respectively (Table 3). For MDA-MB231 cell line, the inhibition percentages of aqueous/methanol, aqueous, n-hexane, and ethyl acetate extracts were 38, 25, 49, and 14%, respectively at concentration of 25 mg/ml (Figures 1A–D). Most extracts were less effective against MDA-MB231 cell line with IC_50_ values more than 25 mg/ml (Table 3). However, n-hexane extract exhibited high activity against this cell line with IC50 value of 3.75 mg/ml.

A dose dependent inhibition for all extracts was also observed in HCT-116, Hela, and VERO cell lines with lower toxicity toward VERO cells (Figure 1 and Table 3).

### Aqueous/Methanol and Aqueous Extracts Have the Highest Phenolic Content

**Figure 2:**
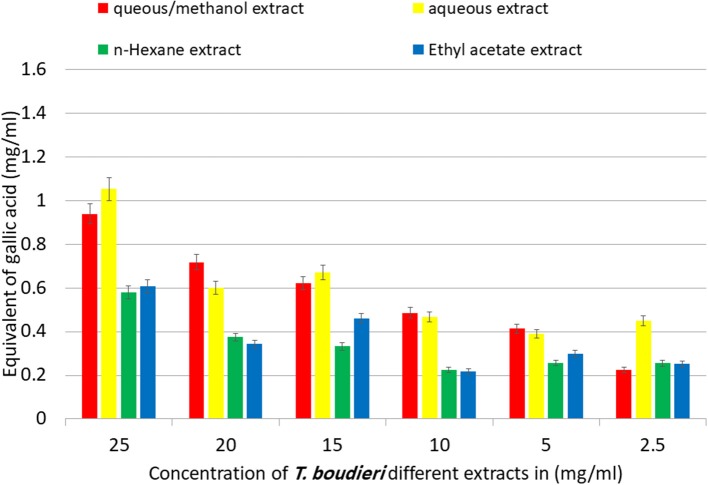
Equivalent of gallic acid in mg per 1 ml of aqueous/methanol, aqueous, n-hexane, and ethyl acetate extracts of *T. boudieri* in different concentrations. Results are expressed as means of three independent experiments (bars) ± SEM (lines).

The TPC in *T. boudieri* extracts was determined according to the Folin-Ciocalteu procedure. The TPC were increasing in a concentration dependent manner. The aqueous/methanol and aqueous extracts showed the highest equivalent of gallic acid with values of 0.94 and 1.05 mg/ml, respectively. On the other hand, n-hexane and ethyl acetate extracts showed lower equivalent of gallic acid with values of 0.58 and 0.61 mg/ml, respectively (Figure 2).

### *T. boudieri* Inhibits Angiogenesis by Inhibiting VEGF Expression

**Figure 3:**
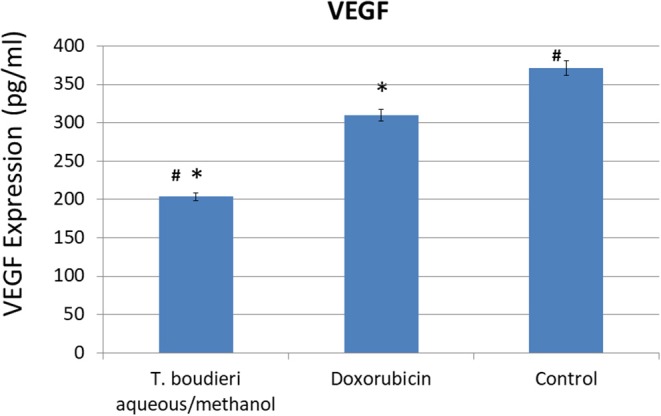
The effect of *T. boudieri* aqueous/methanol extract (at a concentration of 12.5 mg/ml) and doxorubicin (250 nM) on VEGF (vascular endothelial growth factor) expression (pg/ml) in T47D cells. Results are expressed as means of three independent experiments (bars) ± SEM (lines). The asterisks represent significant difference compared with the negative control (*P < 0.05); compared with doxorubicin treatment (^#^*P* < 0.05).

The expression of VEGF was measured *in vitro* in T47D cell line in order to investigate whether the inhibition of angiogenesis may contribute to the observed antiproliferative effect. In the negative control group, VEGF was highly expressed (371 pg/ml). Treating cells with aqueous/methanol extract (12.5 mg/ml) decreased VEGF expression level to 203 pg/ml, while doxorubicin (250 nM) showed a VEGF expression level of 309 pg/ml (Figure 3).

### *T. boudieri* Induces Apoptosis by Increasing Caspase-3 Activity

**Figure 4:**
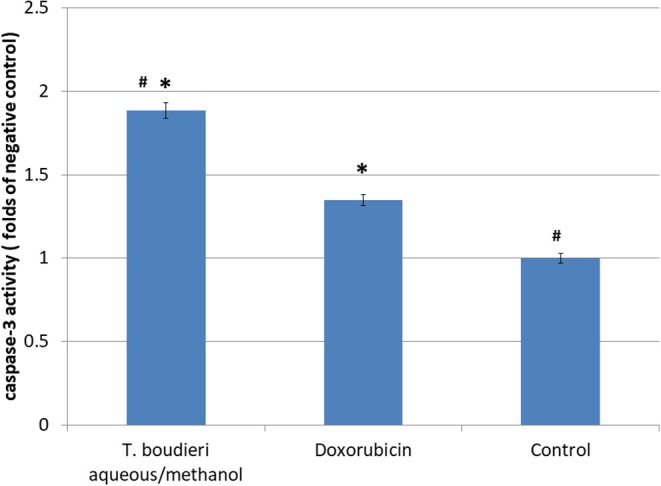
The effect of *T. boudieri* aqueous/methanol extract (at a concentration of 25 mg/ml) and doxorubicin (250 nM) on caspase 3 activity expressed by number of folds of negative control. Results are expressed as means of 3 independent experiments (bars) ± SEM (lines). The asterisks represent significant difference compared with the negative control (*P < 0.05); compared with doxorubicin treatment (^#^*P* < 0.05).

Apoptosis induction in treated cells was measured using caspase-3 activity assay. The aqueous/methanol extract (25 mg/ml) increased caspase-3 activity by 4.85-folds of negative control, while doxorubicin (250 nM) showed an increase of 2.57-folds of the negative control (Figure 4).

### *T. boudieri* Increases the Level of IFN-γ and IL-2

**Figure 5:**
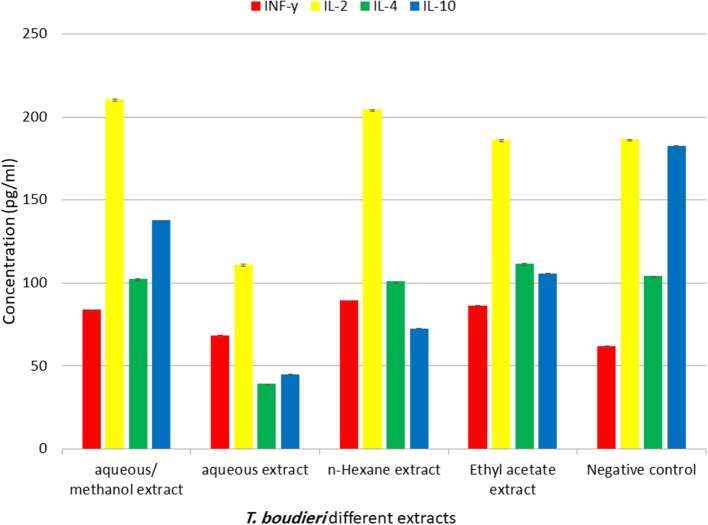
The effect of *T. boudieri* different extracts (at a concentration of 25 mg/ml) on the expression pattern of various cytokines (pg/ml). Results are expressed as means of three independent experiments (bars) ± SEM (lines).

The immuno-modulatory effect of different extracts was evaluated by measuring levels of IFN-γ, IL-4, IL-2, and IL-10 in lymphocytes treated with different solvent extracts of *T. boudieri*. The results indicate that there is significant increase in IFN-γ and IL-2 in lymphocytes treated with 25 mg/ml of different solvent extracts compared with the control group. The highest level of IFN-γ was observed in lymphocytes treated with n-hexane. While aqueous/methanol extract induced the highest increase in (Figure 5).

### *T. boudieri* Stimulates Lymphocytes Proliferation in the Presence and Absence of Mitogens

**Figure 6:**
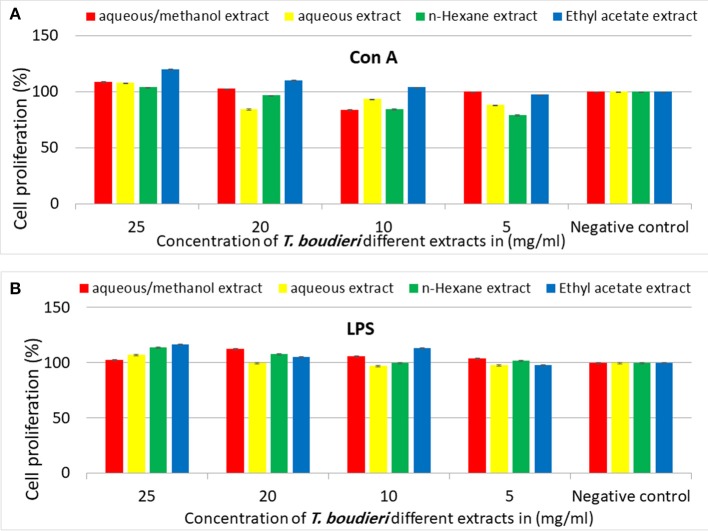
**(A)** The effect of *T. boudieri* different extracts at different concentrations on the proliferation of splenic lymphocytes in the presence of Con A (5 μg/ml). **(B)** The effect of *T. boudieri* different extracts at different concentrations on the proliferation of splenic lymphocytes in the presence of LPS (4 μg/ml). Results are expressed as means of three independent experiments (bars) ± SEM (lines).

**Figure 7:**
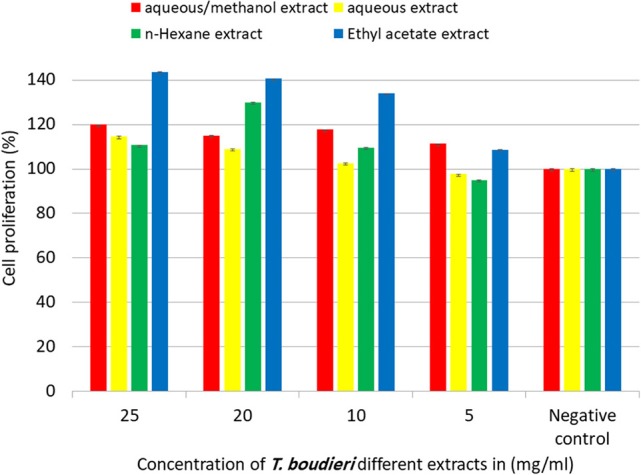
The effect of *T. boudieri* different extracts at different concentrations on the proliferation of splenic lymphocytes in absence of mitogens. Results are expressed as means of three independent experiments (bars) ± SEM (lines).

Our experimental findings showed that most of *T. boudieri* extracts induced an increase in lymphocytes cell proliferation in the presence of Con A and LPS (Figure 6). At a concentration of 25 mg/ml, the most active extract was ethyl acetate with a percentage of cell viability of 120% and 116 on Con A and LPS stimulated cells, respectively. Other solvent extracts showed variable activities (Figure 6). In the absence of mitogenic stimulation, our results showed that all *T. boudieri* extracts induced an increase in lymphocytes cell proliferation with stimulation level reaching 143% for cells treated with ethyl acetate extract (Figure 7).

### *T. boudieri* Enhances Phagocytic and Pinocytic Activity of Peritoneal Macrophages

**Figure 8:**
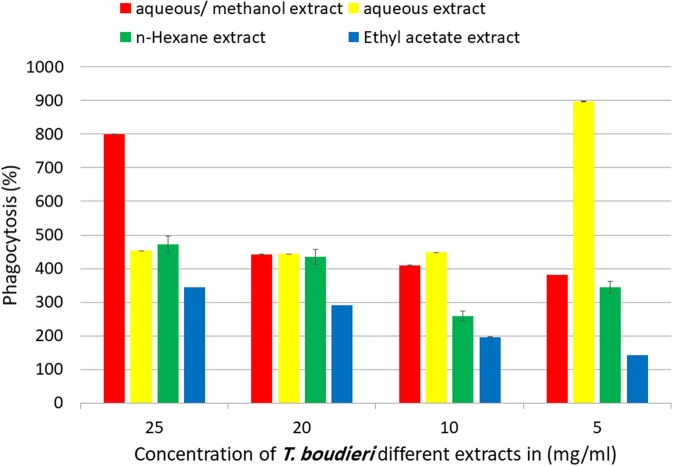
*In vitro* phagocytic assay using nitro blue tetrazolium (NBT) reduction test of peritoneal macrophage treated with various concentrations of *T. boudieri* extracts for 48 h. Results are expressed as means of three independent experiments (bars) ± SEM (lines).

**Figure 9:**
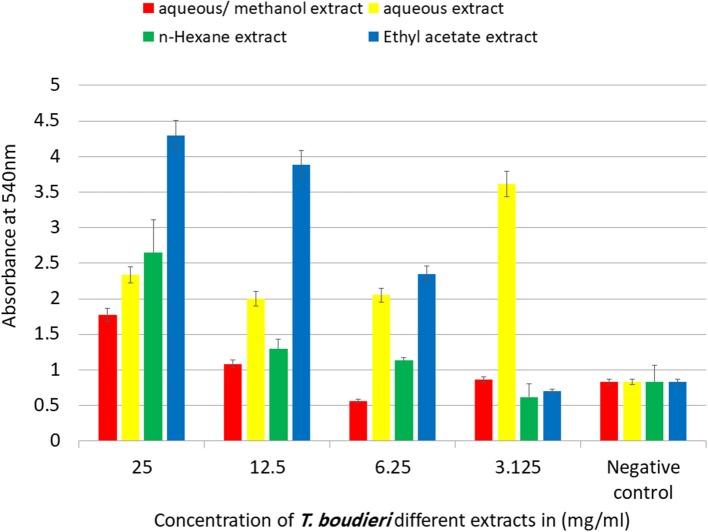
The effect of *T. boudieri* different extracts on macrophage pinocytosis. Results are expressed as means of three independent experiments (bars) ± SEM (lines).

Phagocytic activity of peritoneal macrophages was determined by measuring of NBT reduction ability after treatment with different extracts. Aqueous/methanol extract showed the highest stimulation of peritoneal phagocytic activity at dose of 25 mg/ml (Figure 8). The results of pinocytic assay showed that the effect of most extracts was stimulatory compared with the negative control at the doses ranged from 3.125 to 25 mg/ml. Ethyl acetate extract caused the highest increase in the pinocytic activity with value of compared to control (Figure 9).

## Discussion

The wild edible fungi (WEF) are an important group of non-wood forest products. The history of using WEF is well recorded and highly valued. The biological activities within the fungi kingdom (both truffles and mushrooms) are widely accepted and applied as main component of folk medicine (14).

In our study, we evaluated the anticancer activity and immune modulation effect of *T. boudieri* which is largely used in Middle East as food and to prevent different ailments. Most extracts showed positive results and exhibited immunomodulatory and/or anticancer activities.

In the anticancer part, n-hexane extract was active against all cell lines, while ethyl acetate extract was active against all cell lines except MDA-MB-231. Other solvent extracts showed variable activity against different cell lines (Figure 1). We also noticed a variation in the IC_50_ values of the same extract against different cell lines (Table 3). Previous studies attribute this selectivity to the sensitivity of the cell lines to the nature of the active compounds in these extracts or to the tissue specific response (27, 28).

Studies are very limited for *T. boudieri* anticancer activity. However, many previous studies detected chemical compounds in n-hexane extract of *T. claveryi* which is closely related to *T boudieri*. Campestanol, stigmasterols, and beta-sitosterol are phytosterols found in this extract (29). Phytosterols (naturally occurring steroids) are involved in many mechanisms of action, such as promotion of cancer cells apoptosis, angiogenesis inhibition and inhibiting cancer-cell growth (30).

In our study, the superior activity of n-hexane extract could be explained by the presence of phytosterols which was detected in our phytochemical screening results that showed strong positive result for phytosterol in n-hexane extract (Table 2).

Our results showed a variation of total phenolic content (TPC) from extract to another with a best result for aqueous and aqueous/methanol extracts (Figure 2). Phenolic compounds diminish the initiation, progression, and spread of cancer cells *in vitro* and *in vivo*. The mechanisms that phenolics modulate to achieve these anticancer effects are multi-faceted, including cell signaling cascades and regulation of growth factor-receptor interactions, which involve transcription factors and kinases, that determine genes expression involved in cell survival, cell cycle arrest and apoptosis (31).

All extracts were not active against MDA-MB231 cell line except n-hexane extract which showed only a mild activity. This may be explained by the features of MDA-MB231 cell line, which is characterized by the absence of the expression of ER (oestrogen receptor), PR (progesterone receptor), and HER2 (human epidermal growth factor receptor 2), sometimes it is mentioned as triple-negative. The lack of the expression of a recognized therapeutic target, makes MDA-MB231 more biologically aggressive, difficult to treat and often have a poor prognosis (32). On the other hand, all of the extracts showed low activity against Vero normal cells as indicated by high IC_50_ values, this may indicate safety of these extracts against normal cell (Table 3).

Vascular endothelial growth factor (VEGF) is a protein that stimulates the formation of blood vessels (angiogenesis process). This protein is an important target in cancer treatment to inhibit cancer cells proliferation by the prevention of blood vessels formation (33). Aqueous/methanol extract exhibited high activity to inhibit angiogenesis, compared to doxorubicin (positive control) (Figure 3). The phytochemical test showed positive results for phenols in aqueous/methanol extract. This result agrees with previous studies which confirm the potential activity of phenols in decreasing the risk of developing many types of cancer and decreasing the risk of cancer progression, due to their ability to decrease vascular endothelial growth factor (VEGF) expression and cell viability (34).

The induction of apoptosis is another mechanism responsible for anticancer activity. It is highly organized and controlled process and helps the body in maintaining its homeostasis (35). In cancer, this process is not working properly due to induction of the anti-apoptotic gene and inhibition of the pro-apoptotic gene thus the cell division and proliferation will continue (36). In our study, the aqueous/methanol extract of *T. boudieri* induced apoptosis by enhancing caspase-3 activity. Such result could be explained by the presence of phenols, steroids, terpenoids, and carbohydrates in this extract. These compounds exhibit clear apoptosis induction ability as indicated by previous studies (37, 38).

The immune system is a complex defense system in vertebrates. It contains a sophisticated network of cells, tissues and organs that work together to defend the body from foreign invaders such as bacteria, fungi, and parasites that can cause infections (39).

In our study, variations in immune response due to exposure to truffle extracts were also explored through measuring levels of IFN-γ, IL-2, IL-4, and IL-10. The current results indicate that there is an up-regulation in IFN-γ and IL-2 in lymphocytes treated with some truffle extracts (Figure 5). High levels of IFN-γ and IL-2 indicates stimulation of Th1 immune response which is important in anticancer immune response. On the other hand, high IL-4 stimulates Th2 immune response (40). Our results showed that different solvent extracts of *T. boudieri* can stimulate the immune system toward Th1. N-hexane extract showed the highest effect, followed by ethyl acetate and aqueous/methanol extracts (Figure 5). Many studies mentioned the immunomodulatory effect of truffle species (41, 42), and this confirm our results. This effect may be explained by the presence of different phytochemicals in truffle extracts.

Lymphocytes proliferation assay was conducted to measure the effect of each extract on lymphocytes proliferation and activation. Ethyl acetate extract showed the highest effect as a stimulator of lymphocytes (Figures 6, 7). This extract is rich in terpenoids which may explain its activity. A previous study showed that monoterpene enhances splenocytes proliferation in Balb/C mice (43).

Phagocytic activity test and pinocytic activity test were conducted to evaluate the effect of each extract on innate immunity. Macrophages play an important role in phagocytosis which is responsible for the intracellular killing of antigen and other apoptotic cells. Therefore, the modulation of macrophage activity seems to play a chief role in regulation of innate immunity (44). The aqueous/methanol extract showed the highest stimulation, followed by n-hexane, aqueous, and ethyl acetate extracts (Figures 8, 9). The high activity of aqueous/methanol extract may be explained by the presence of flavonoids in these extracts, as mentioned previously in our phytochemical tests. A previous study on flavonoids indicates that they can enhance macrophage function (45).

## Conclusion

*Terfezia boudieri* is a truffle rich in biologically active phytochemicals. It contains compounds with anticancer and immunomodulatory effects. The anticancer activity of its extracts is mediated by angiogenesis inhibition and apoptosis induction. The immunomodulatory effect is mediated by activation of innate and acquired arms of the immune system. Further studies are needed to isolate and identify pure active compounds from this truffle to fully understand its anticancer and immunomodulatory effects.

## Data Availability Statement

The datasets generated for this study are available on request to the corresponding author.

## Author Contributions

The project idea was developed by WT. The experimental design was developed by WT and WH. Sample collections were performed by MA. The laboratory experiments were run by MA. The data were analyzed by WT, LA, and MA. The manuscript was written and revised by WT, LA, and MA.

### Conflict of Interest

The authors declare that the research was conducted in the absence of any commercial or financial relationships that could be construed as a potential conflict of interest.
